# TTK is a potential regulator of tumor progression correlated with dedifferentiation and immune cell infiltration in papillary thyroid cancer

**DOI:** 10.18632/aging.205100

**Published:** 2023-10-06

**Authors:** Jun-Jie Ma, Cheng Xiang, Jian-Wei Wang

**Affiliations:** 1 Department of Thyroid Surgery, The Second Affiliated Hospital of Zhejiang University School of Medicine, Hangzhou 310009, Zhejiang, China; 2 The Colorectal Surgery, The Second Affiliated Hospital of Zhejiang University School of Medicine, Hangzhou 310009, Zhejiang, China

**Keywords:** TTK, papillary thyroid cancer, tumorigenesis, prognosis, biomarker

## Abstract

Objective: To investigate the role and clinical significance of threonine tyrosine kinase (TTK) in papillary thyroid cancer (PTC).

Methods: TTK expression in PTC and normal groups were compared using TCGA data and *in vitro* experiments. The prognostic value of TTK and its possible role in PTC dedifferentiation was evaluated. Next, TTK involvement in PTC occurrence and progression was analyzed via *in vitro* experiments. Subsequently, analyses of enrichment and immune cell infiltration were conducted to reveal the possible mechanism. Finally, we predicted the target miRNAs followed by performing a luciferase reporter experiment.

Results: TTK upregulation was observed in PTC, and its elevated level was significantly related to an unfavorable prognosis (*P* < 0.05). Interestingly, TTK negatively correlated with thyroid differentiation score (TDS), and patients with higher TDS showed longer survival (all *P* < 0.05). PTC cell growth, migration, and invasion were inhibited upon TTK knockdown. Besides, TTK was involved in metabolic processes and regulated cell adhesion molecules pathway. Its overexpression was positively associated with immune cell infiltrates (*P* < 0.05). Moreover, miR-582-5p was an upstream target of TTK.

Conclusion: TTK serves as a potential biomarker for tumorigenesis and prognosis in PTC, especially for those that may differentiate into more aggressive thyroid cancers.

## INTRODUCTION

Thyroid cancer (THCA) is the most prevalent malignancy of the endocrine system, of which morbidity has been increasing worldwide [1] Its incidence ranked 9th leading cancer among malignancies globally in 2020 [2, 3]. Papillary thyroid cancer (PTC) accounts for 80–90% of all THCA cases and tends to metastasize to the cervical lymph nodes [4, 5]. Fine-needle aspiration cytology is a gold standard diagnosis of PTC; however, the rate of indeterminate lesions offers a challenge in clinical management [6, 7]. Although the vast majority of patients with PTC have a favorable 5-year survival rate of over 95% after standard surgery, radioactive iodine therapy, and thyroid-stimulating hormone suppression therapy, 5–10% of them still experience recurrence or distant metastasis since some PTC dedifferentiate into more aggressive phenotypes [8, 9]. Multiple factors contribute to the initiation and development of PTC, including epigenetic alterations, environmental exposures, and genetic mutations [10]. Epigenetic alterations include Linc00941, MiR21, and MiR146b and genetic mutations include the driver point mutations of RAS and BRAF [11, 12]. Some of these markers have been used in the clinic as diagnostic markers and therapeutic targets for recurrence and metastasis, but there are still significant limitations in diagnostic efficiency and treatment side effects [13]. Therefore, it is necessary for robust molecular biomarkers to be developed for improving the diagnosis and treatment management of PTC.

Threonine tyrosine kinase (TTK) also known as Mps1, is a dual-specificity kinase that phosphorylates tyrosine as well as serine/threonine [14]. In addition to promoting mitotic checkpoint complex formation, TTK regulates cytokinesis, responds to DNA damage, and facilitates proper chromosome alignment [15]. As a mediator of the spindle assembly checkpoint (SAC), TTK delays anaphase until all chromosomes are properly attached to the mitotic spindle, which ensures accurate chromosomal segregation and preserves genomic integrity [16]. Since most cancer cells are aneuploidy, they rely heavily on SACs to adequately segregate their abnormal karyotypes during mitosis [17]. Thus, TTK promotes cancer cell proliferation and invasion, and its upregulation leads to poor prognosis for various cancers of the breast, colon, and glioblastoma [17–19]. However, the impact of TTK on PTC progression and its underlying mechanism remains to be clarified.

Herein, we used efficient bioinformatics tools to analyze the TTK expression and validated it in PTC and normal thyroid cells. After examining the clinical value, we assessed its influence on cancer cell proliferation, apoptosis, cell cycle, invasion, and migration. Then, the biological processes and gene set enrichment analysis (GSEA) was conducted to explore the potential regulatory pathway of TTK, which was validated using *in vitro* experiments. Immune cell infiltration analysis was performed using the TCGA data. Finally, the upstream miRNAs were predicted to further determine the fundamental mechanism.

## MATERIALS AND METHODS

### TTK expression analysis

TIMER (https://cistrome.shinyapps.io/timer/) was employed to analyze differential TTK expression in pan-cancer in the “Diff Exp” column. Then, UCSC Xena (https://xenabrowser.net/) was searched to extract the expression and clinical data of TCGA-THCA. PTC samples exhibiting complete expression data matched with survival information were included. Unpaired and paired *t*-tests were used for analyzing TTK expression in PTC and normal groups.

Besides, all participants were assigned to low (TTK-L) and high expression groups (TTK-H) by the median TTK level. The correlation of TTK expression as a categorical variable and the clinical factors was evaluated using the chi-square test, and univariate logistic regression analysis. As a continuous variable, the correlation with these parameters was analyzed by the Student’s *t*-test or one-way ANOVA.

### Clinical significance of TTK in PTC

Firstly, the receiver operating characteristic (ROC) curves were drawn to determine the diagnostic value of TTK in PTC. Next, the effect of TTK on clinical outcomes was analyzed by the Kaplan-Meier plotter method. Disease-free interval (DFI) refers to the measure of time after treatment during which no regional recurrence/distant metastasis is found [20]. Disease-specific survival (DSS) refers to the percentage of people who died from a specific disease in a defined period [21]. Overall survival (OS) is defined as the time from the operation to death from any cause [22]. Progression-free interval (PFI) was measured from the date of initial treatment randomization to the time of disease recurrence [23].

Following this, its predictive value for survival status was evaluated using the R package “timeROC”. The Cox regression analyses were conducted to obtain the independent risk factors for PTC. Finally, nomogram models were constructed and the calibration curves were plotted.

Subsequently, the association of TTK expression with thyroid differentiation score (TDS) was assessed using the Pearson correlation test to explore the value of TTK in PTC dedifferentiation. The mRNA expression level of 16 thyroid-specific genes was used to calculate the TDS. Then, a median TDS level was used to divide the patients into high- and low-TDS groups. The correlation of TDS with DFI and PFI was assessed by the Kaplan-Meier plotter method.

### Cell lines and cell culture

Normal thyroid cell line Nthy-ori 3–1 and PTC cell lines TPC1 and BCPAP were used in this study (Shanghai, China). The cells were cultured in Dulbecco’s modified Eagle’s medium (Gibco, Waltham, MA, USA) supplemented with 10% fetal bovine serum and 100 units/ml of penicillin-streptomycin (Gibco, USA) at 37°C in a humidified atmosphere of 5% CO_2_.

### Quantitative real-time PCR (qRT-PCR)

Total RNA was isolated from cells using TRIzol reagent (Invitrogen, Carlsbad, CA, USA). The supernatant was transferred after centrifugation at 12 000 g at 4°C for 10 min and was placed at room temperature for 5 min, added with 0.2 ml chloroform, vibrated for 15 s, and placed at 37°C for 2–3 min. After the water phase was transferred and the supernatant removed, the RNA was stored at −70°C. Following reverse transcription, qRT-PCR was performed on an Mx3000P system (Agilent Stratagene). GAPDH was utilized as an internal control. Relative expressions of TTK and miR-582-5p were measured using the 2^−ΔΔCt^ method. The primer sequences are shown in Table 1.

**Table 1:** Primer sequences.

**Name**	**Primer sequences**
Human TTK forward	5′-CCGAGATTTGGTTGTGCCTGGA-3′
Human TTK reverse	5′-CATTCGACACCAGAGGTTCCTTG-3′
Human miR-582-5p forward	5′-GCACACATTGAAGAGGACAGAC-3′
Human miR-582-5p reverse	5′-TATTGAAGGGGGTTCTGGTG-3′
Human GAPDH forward	5′-GAAGGTGAAGGTCGGAGTC-3′
Human GAPDH reverse	5′-GAAGATGGTGATGGGATTTC-3′

### Western blotting

Cells were lysed with RIPA lysis buffer (Beyotime, Shanghai, China) and the protein concentration was quantified by the BCA method. Then, the proteins were dissolved by sodium dodecyl sulfate-polyacrylamide gel electrophoresis and were transferred to polyvinylidene fluoride (PVDF) membranes (Beyotime, Shanghai, China), blocked with 5% skimmed milk. The membranes were incubated with the following primary antibodies at 4°C overnight: anti-TTK (Abcam), anti-GAPDH (Abcam), anti-Bcl2 (Abcam), anti-Bax (Abcam), anti-CDK2 (Abcam), anti-Cyclin D1 (Abcam), anti-p21 (Abcam), anti-E-cadherin (Abcam), anti-N-cadherin (Abcam), anti-Vimentin (Abcam), anti- MMP2 (Abcam), anti-Snail (Abcam), anti-Twist1 (Abcam), anti-ICAM1 (Abcam), anti-VCAM1 (Abcam), anti-MMP9 (Abcam). The next day, after incubation with a goat anti-rabbit IgG secondary antibody (Abcam), the PVDF membrane was subjected to chemiluminescence detection by an ECL western blotting detection kit (Servicebio, Wuhan, China).

### Cell transfection

Eukaryotic expression vectors encoding TTK and plasmids encoding shRNAs against TTK were synthesized by GenePharma (Shanghai, China). Next, the PTC cells were transfected with shRNA plasmids according to the manufacturer’s instructions. The transduced cells were selected with 2 μg/mL puromycin for 4 weeks. Lentiviral particles packaging the shRNA are targeting TTK #1 (5′-AATGAACAAAGTGAGAGACAT-3′), TTK #2 (5′-UGAACAAAGUGAGAGACAUTT-3′), TTK #3 (5′-GGAUUUAAGUGGCAGAGAGAATT-3′), and the scramble control (5′-TTCTCCGAACGTGTCACGT-3′).

### CCK8 and colony formation assays

CCK8 assay was utilized to monitor cell growth by the manufacturer’s protocols. Cells were seeded in 96-well plates (5 × 10^3^ cells/well) and CCK8 solution (10 μL) was added to each well. Next, a microplate reader was used to measure the absorbance at 450 nm after incubation of the cells at 37°C for 2 h. Colony formation assay was conducted for verifying how TTK affects PTC cell proliferation. PTC cells transfected with shRNA plasmids were seeded into well of 6-well plates and cultured for 2–3 weeks until colony formation; fresh media was provided every 3 days. Finally, to fix the colonies with paraformaldehyde (1 ml/well), stain them with crystal violet solution (1 ml/well), and quantify them.

### Flow cytometry assay

Flow cytometry assay was conducted as previously described [24]. Firstly, cells were resuspended in binding buffer (300 μL) after being washed twice, and then Annexin V-FITC solution (5 μL) was added for incubation in the dark for 15 min. Following propidium iodide (PI) (10 μL) supplementation and culture for 30 min, the apoptotic cells were evaluated by an Annexin V-FITC/PI Apoptosis Detection kit (BD Biosciences, Franklin Lakes, NJ, USA). Additionally, the PTC cells during the exponential growth phase were harvested, and single-cell suspensions containing 1 × 10^5^ cells were fixed with 70% alcohol. Then, the cell cycle analysis was performed with a FACScan flow cytometer, and the data were analyzed using FlowJo software.

### Transwell assay

Transwell assay was utilized to evaluate the invasive capacity of the cells. The cells in the logarithmic growth phase were trypsinized, resuspended in a serum-free medium, and adjusted to a density of 1 × 10^6^ cells/mL. Cell suspension (200 μL) and 600 μL medium were added to the top and bottom compartments, respectively, and then the cells were cultured at room temperature for 24 h. Following this, cells migrating to the bottom surface were fixed with methanol and dyed with 0.1% crystal violet at 37°C in the dark for 30 min. After rinsing the stained cells with PBS three times, they were observed through a light microscope.

### Wound-healing assay

We seeded the cells into 6-well plates (5 × 10^5^ cells/well). When the cells reached 90% confluence, a sterile 200-μL pipet tip was used to produce a wound line in the center of the cell monolayer. We took the wound images and recorded the width at 0 h after rinsing two times with PBS. The image of the same location was obtained after 24 h. The cellular migration ability was assessed by measuring changes in scratch space distance.

### Enrichment analysis

We first identified differentially expressed genes (DEGs) by the median TTK expression using the “limma” package. The thresholds were set as follows: *P* < 0.05 and |log 2 (Fold Change)|>1. Then, the biological processes of these significant DEGs were analyzed by the “ClusterProfiler” package.

Subsequently, GSEA was performed using the TCGA data. By setting the median as the cutoff value, patients were divided into TTK-H and TTK-L. The number of gene set permutations was 1000 times and a phenotypic label was set as high expression versus low expression.

### Immune cell infiltration analysis

Tumor-infiltrating immune cells play an essential role in tumor occurrence, progression, and metastasis. Thus, the immune score and stromal score were calculated using the ESTIMATE algorithm [25]. Besides, the TIMER and xCELL algorithms were adopted to evaluate the expression correlation of TTK with several immune cells.

### Luciferase reporter experiment

We predicted the target miRNAs of TTK through DIANA (http://diana.imis.athena-innovation.gr/DianaTools/index.php), miRDB (http://www.mirdb.org/), and Targetscan (http://www.targetscan.org/vert_72/) databases. The Venn analysis identified nine consistent miRNAs among the three databases and miR-582-5p of interest was selected for further luciferase reporter experiment [26]. Then, the binding sequence was acquired by the bioinformatics tools, and PCR was implemented to amply the binding site. The TTK 3′UTR wild-type and mutant plasmids were constructed followed by co-transfecting the recombinant plasmids into TPC1 and BCPAP cells with miR-NC and miR-582-5p mimics, respectively. A dual-luciferase reporter gene assay system was used for luciferase activity determination after 48 h.

### Statistical analysis

Data analyses were conducted by GraphPad Prism software (version 8.0.2, San Diego, CA, USA), SPSS software (version 23.0), and R software. Data from the cellular experiments were presented as mean ± standard deviation. Student’s *t*-test was used for two-group comparison, while a one-way ANOVA was adopted to determine the differences for at least three groups. All experiments were repeated at least three times and *P* < 0.05 was defined as statistically significant.

## RESULTS

### TTK is overexpressed in PTC

**Figure 1:**
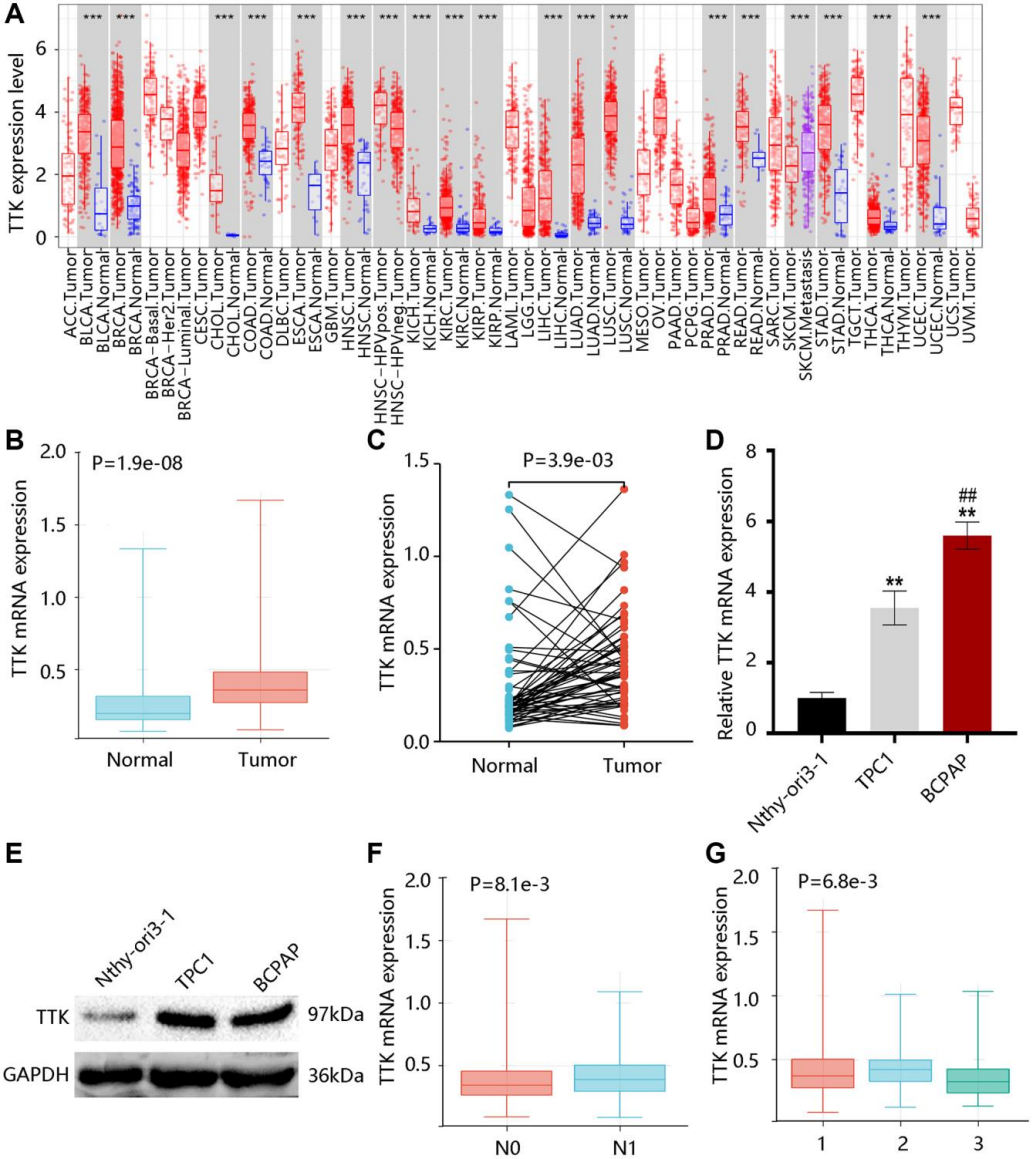
**TTK expression profile in papillary thyroid cancer (PTC).** (**A**) TTK expression in pan-cancer. The elevated TTK mRNA level in PTC by the (**B**) unpaired and (**C**) paired *t*-tests using TCGA data. Higher TTK (**D**) mRNA and (**E**) protein expression in PTC cells. Relationship between TTK and (**F**) N stage, as well as (**G**) histological subtype. 1: PTC-classical; 2: PTC-tall cell; 3: PTC-follicular.

TTK transcriptional levels in various cancers were exhibited in Figure 1A. Of note, higher TTK expression was found in PTC in contrast with the normal group (all *P* < 0.05) (Figure 1B, 1C). Moreover, its mRNA level in the PTC cell was significantly higher in contrast with the normal cell, while the BCPAP cell had a notably higher level than the TPC1 cell (*P* < 0.05) (Figure 1D). Besides, we observed the elevated TTK protein level of PTC cells compared with the normal cell (Figure 1E). These results indicated that TTK was upregulated in PTC.

After that, we examined that TTK was remarkably linked to the N stage and histological subtype but was not connected with age and gender (Tables 2, 3). Notably, TTK expression was elevated in the N1 stage; and its expression was upregulated in the PTC-tall cell subtype, followed by PTC-classical and PTC-follicular (*P* < 0.05) (Figure 1F, 1G).

**Table 2:** The correlation between TTK expression and clinicopathological parameters in PTC.

**Parameters**	**Total *n* (%)**	**Low TTK *n* (%)**	**High TTK *n* (%)**	***P*-value**
Age				
<55	337 (67.1)	171 (68.1)	166 (66.1)	0.635
≥55	165 (32.9)	80 (31.9)	85 (33.9)	
Gender				
Female	366 (72.9)	180 (71.7)	186 (74.1)	0.547
Male	136 (27.1)	71 (28.3)	65 (25.9)	
N stage				
N0	225 (44.8)	125 (49.8)	100 (39.8)	0.013
N1	228 (45.4)	100 (39.8)	128 (51.0)	
Unknown	49 (9.8)	26 (10.4)	23 (9.2)	
Subtype				
PTC-classical	361 (71.9)	174 (69.3)	187 (74.5)	0.010
PTC-tall cell	37 (7.4)	13 (5.2)	24 (9.6)	
PTC-follicular	104 (20.7)	64 (25.5)	40 (15.9)	

Abbreviation: PTC: papillary thyroid cancer.

**Table 3:** The connection of TTK with clinical factors through univariate logistic regression analysis.

**Characteristics**	**Odds ratio (95% CI)**	***P*-value**
Age	0.995 (0.984–1.006)	0.374
Gender	0.886 (0.597–1.314)	0.547
N stage	1.600 (1.104–2.318)	0.013
Subtype	0.800 (0.644–0.993)	0.043

Abbreviation: 95% CI: 95% confidence interval.

### TTK overexpression is related to unfavorable prognosis

**Figure 2:**
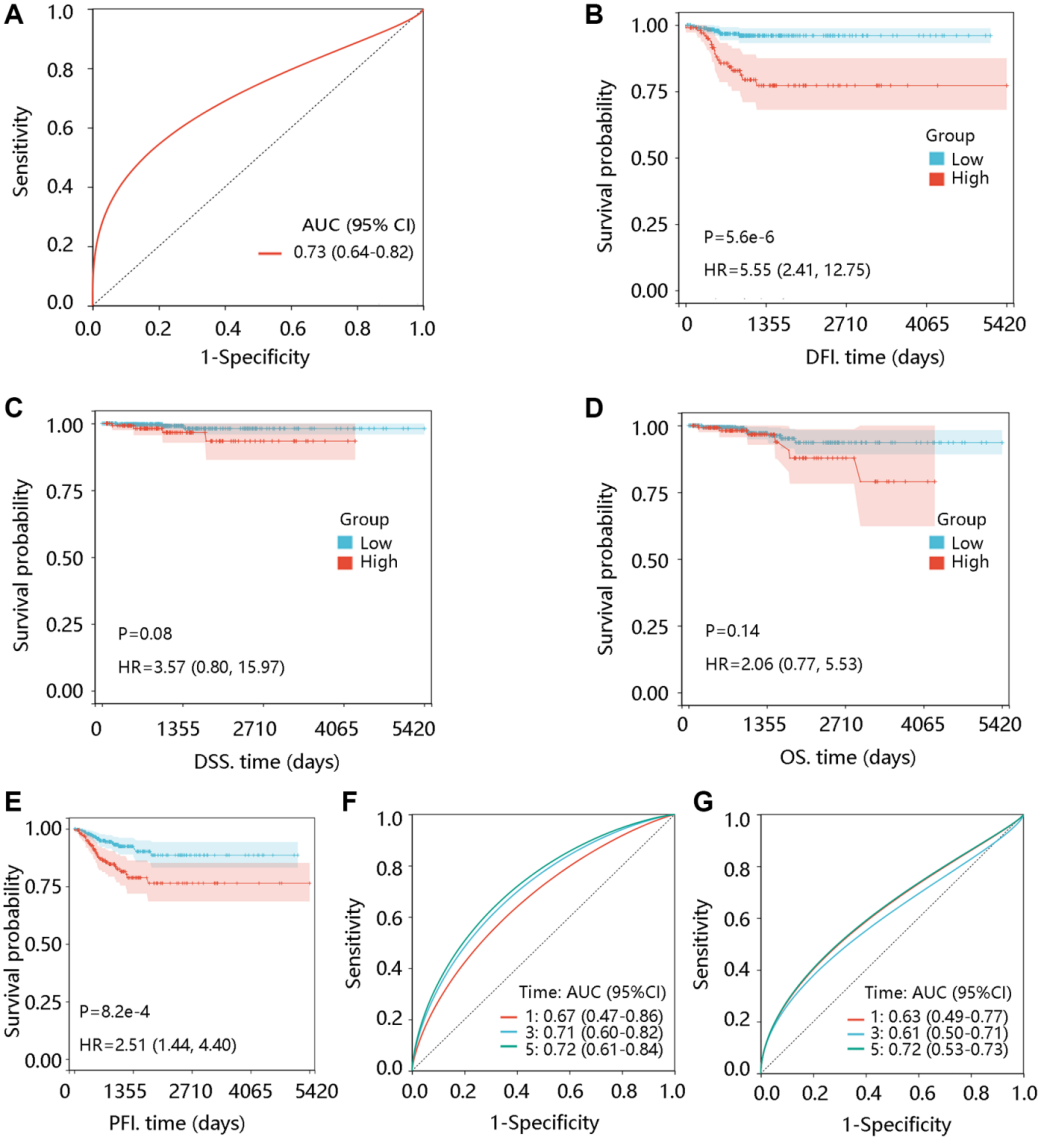
**Prognostic significance of TTK in PTC.** (**A**) Value of TTK in distinguishing the PTC and normal tissues. The association of TTK with (**B**) disease-free interval (DFI), (**C**) disease-specific survival (DSS), (**D**) overall survival (OS), and (**E**) progression-free interval (PFI). TTK showed a certain ability in predicting the (**F**) DFI and (**G**) PFI status. Abbreviations: AUC: area under the curve; HR: hazards ratio; 95% CI: 95% confidence interval.

The ROC curve was plotted to investigate the significance of TTK in discriminating PTC and normal thyroid tissue with an area under the curve of 0.73 (Figure 2A). In addition, TTK upregulation was closely connected with shorter DFI and PFI (*P* < 0.05) but had no significant association with DSS and OS (*P* > 0.05) (Figure 2B–2E). Moreover, TTK had a certain ability in predicting DFI and PFI status (Figure 2F, 2G).

**Figure 3:**
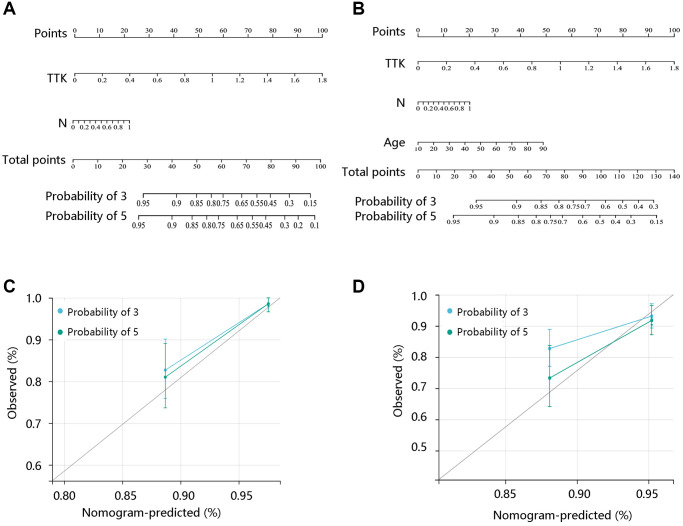
**Construction and evaluation of nomogram models.** Nomogram model construction by integrating the independent predictors for (**A**) DFI and (**B**) PFI. Evaluation of the nomogram models based on the (**C**) DFI and (**D**) PFI.

To reveal the independent prognostic role of TTK, we performed the Cox regression analysis of DFI and PFI. In the univariate analysis, N stage and TTK were significantly related to DFI. The significant factors were then enrolled for multivariate analysis and they remained independent predictors (*P* < 0.05). Moreover, age, N stage, and TTK could independently predict the PFI (*P* < 0.05) (Table 4). Among these independent predictors, TTK contributed most to predicting the DFI and PFI probability of 3 and 5 years (Figure 3A, 3B). The calibration curves showed good agreement between the prediction by the nomogram and actual observations (Figure 3C, 3D). These findings implied TTK as a potent prognostic predictor.

**Table 4:** Cox regression analysis of TTK and clinical factors for disease-free interval and progression-free interval.

**Characteristics**	**Univariate analysis**		**Multivariate analysis**	
	**HR (95% CI)**	***P* value**	**HR (95% CI)**	***P*-value**
**Disease-free interval**				
Age	0.992 (0.968–1.018)	0.552	/	/
Gender	1.286 (0.540–3.060)	0.570	/	/
N stage	3.090 (1.271–7.513)	0.013	3.381 (1.360–8.405)	0.009
Subtype	0.728 (0.417–1.273)	0.266	/	/
TTK	15.399 (5.421–43.745)	<0.001	19.488 (5.598–67.846)	<0.001
**Progression-free interval**				
Age	1.019 (1.003–1.036)	0.024	1.020 (1.003–1.037)	0.018
Gender	1.660 (0.943–2.920)	0.079	/	/
N stage	1.850 (1.024–3.343)	0.042	1.926 (1.063–3.490)	0.031
Subtype	0.781 (0.530–1.152)	0.213	/	/
TTK	8.449 (3.263–21.876)	<0.001	6.084 (2.173–17.031)	0.001

Abbreviations: HR: hazard ratio; 95% CI: 95% confidence interval.

### TTK negatively correlates with TDS

**Figure 4:**
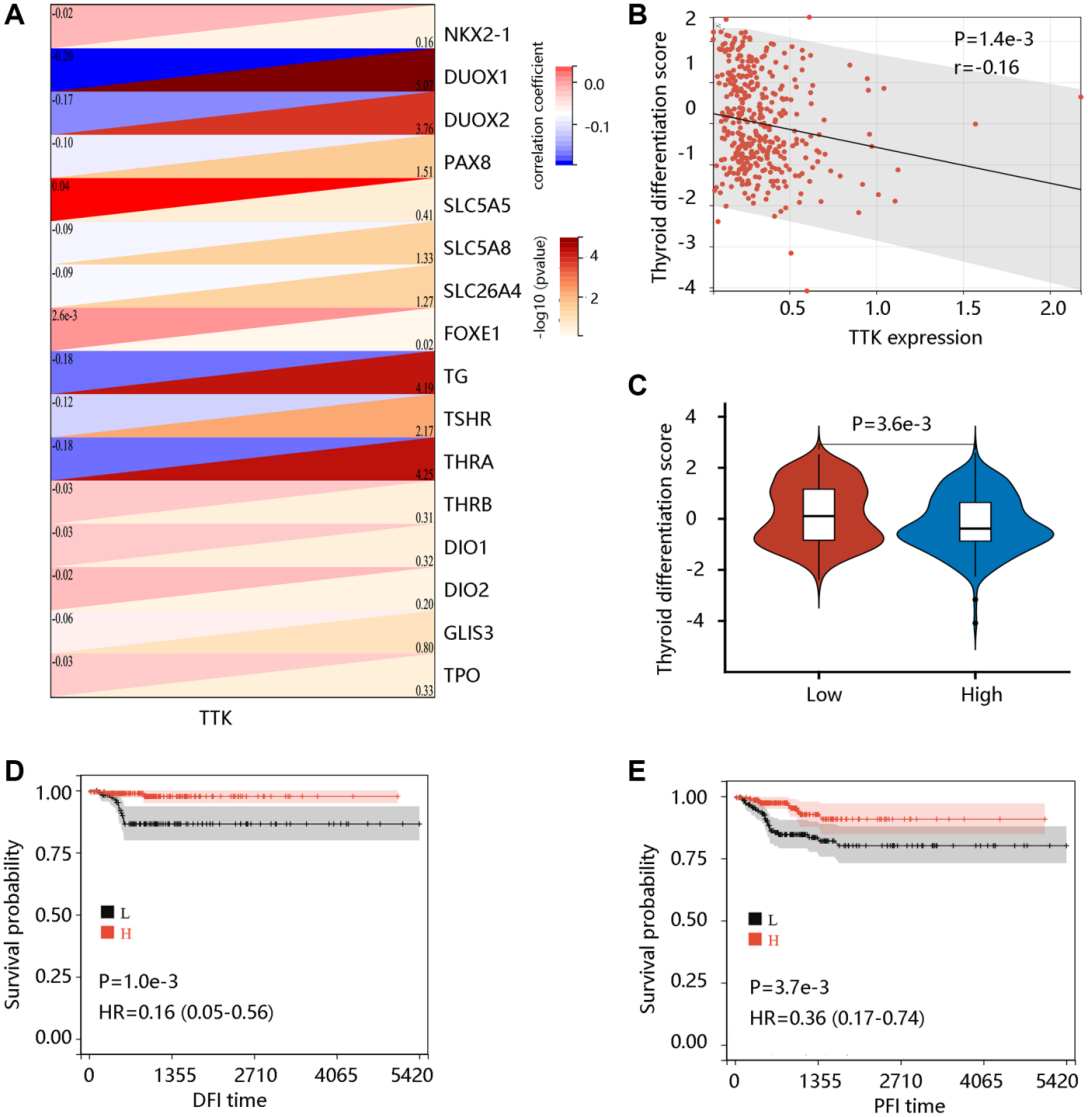
**The value of TTK in PTC dedifferentiation.** (**A**) Significant association of TTK with 16 thyroid-specific genes. (**B**) The significant negative correlation between TTK and thyroid differentiation score (TDS). (**C**) Higher TDS in low TTK expression group. Patients with high TDS had favorable (**D**) DFI and (**E**) PFI.

To examine the potential role of TTK in PTC dedifferentiation, we analyzed the correlation of TTK expression with TDS. TTK was negatively correlated with the expression of 16 thyroid-specific genes (Figure 4A). TTK had a negative correlation with TDS, and patients in the TTK-H presented markedly lower TDS (all *P* < 0.05) (Figure 4B, 4C). Interestingly, high TDS was significantly related to favorable DFI and PFI (all *P* < 0.05) (Figure 4D, 4E). These results suggested that TTK might affect patient prognosis by influencing PTC dedifferentiation.

### TTK promotes PTC cell growth

**Figure 5:**
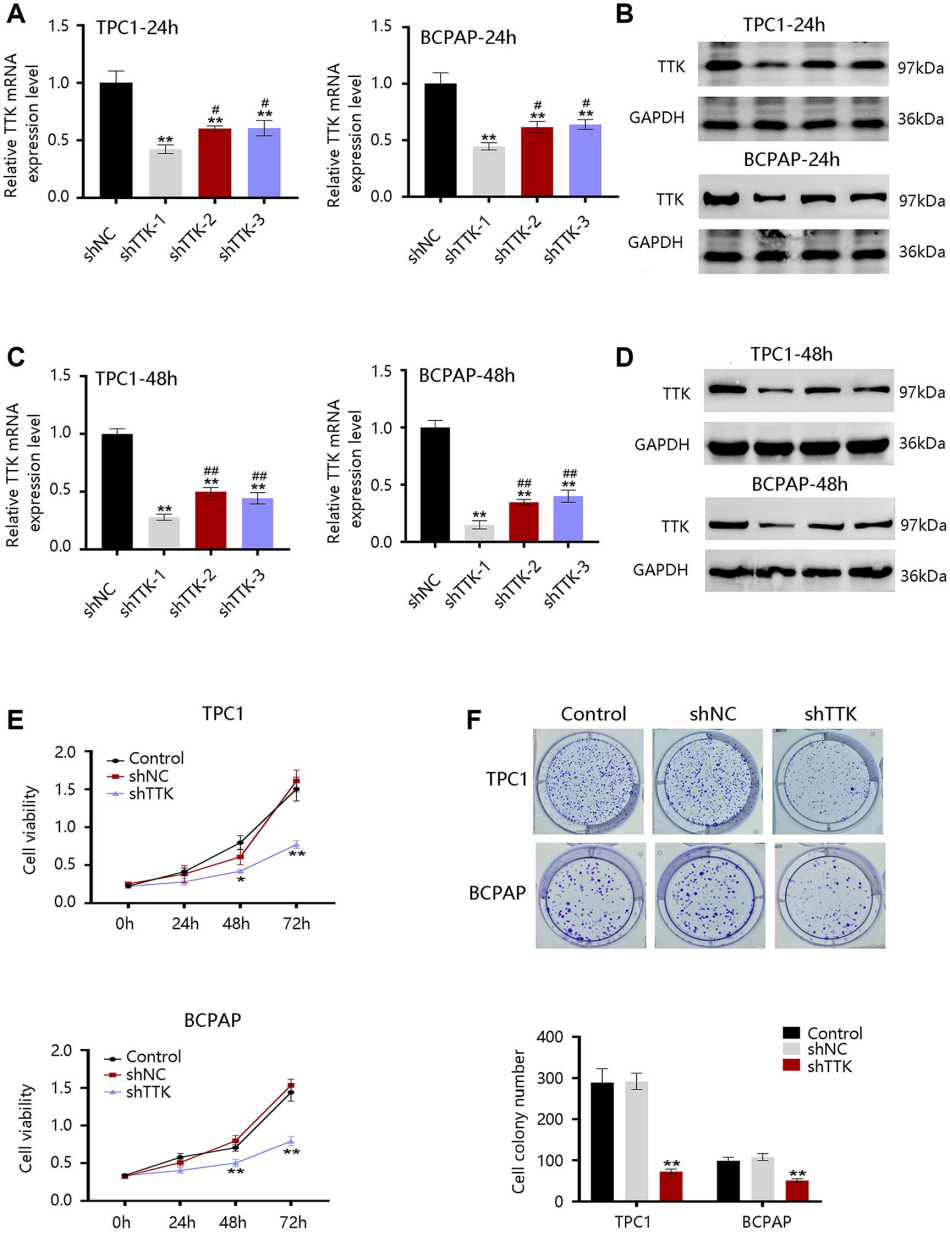
**Effect of TTK on PTC cell growth.** TTK knockdown efficiency of cells transfected with scrambled shRNA (shNC) or shRNAs against TTK (shTTK) for (**A**, **B**) 24 h and (**C**, **D**) 48 h in TPC1 and BCPAP cells. (**E**) The viability of TPC1 and BCPAP cells following TTK knockdown. (**F**) Representative images (up) and quantification (down) of colony formation assays of two cells. Compared with shNC, ^*^P < 0.05, ^**^P < 0.01; compared with shTTK-1, ^#^*P* < 0.05, ^##^*P* < 0.01.

The TTK overexpression and its clinical value in PTC promoted us to investigate its oncogenic effect in PTC. TTK was knocked down in TPC1 and BCPAP cells using TTK-targeting shRNA vectors. We selected shRNA-1 for all following experiments due to its highest knockdown efficiency as determined by the qRT-PCR and western blotting results (Figure 5A–5D). The TTK-knockdown TPC1 and BCPAP cells significantly suppressed cell proliferation as exhibited by CCK-8 (Figure 5E). Consistent findings were observed in the colony formation assay (Figure 5F). These data indicated that TTK might accelerate PTC cell growth.

### TTK promotes PTC cell growth by inhibiting apoptosis and facilitating cell cycle progression

**Figure 6:**
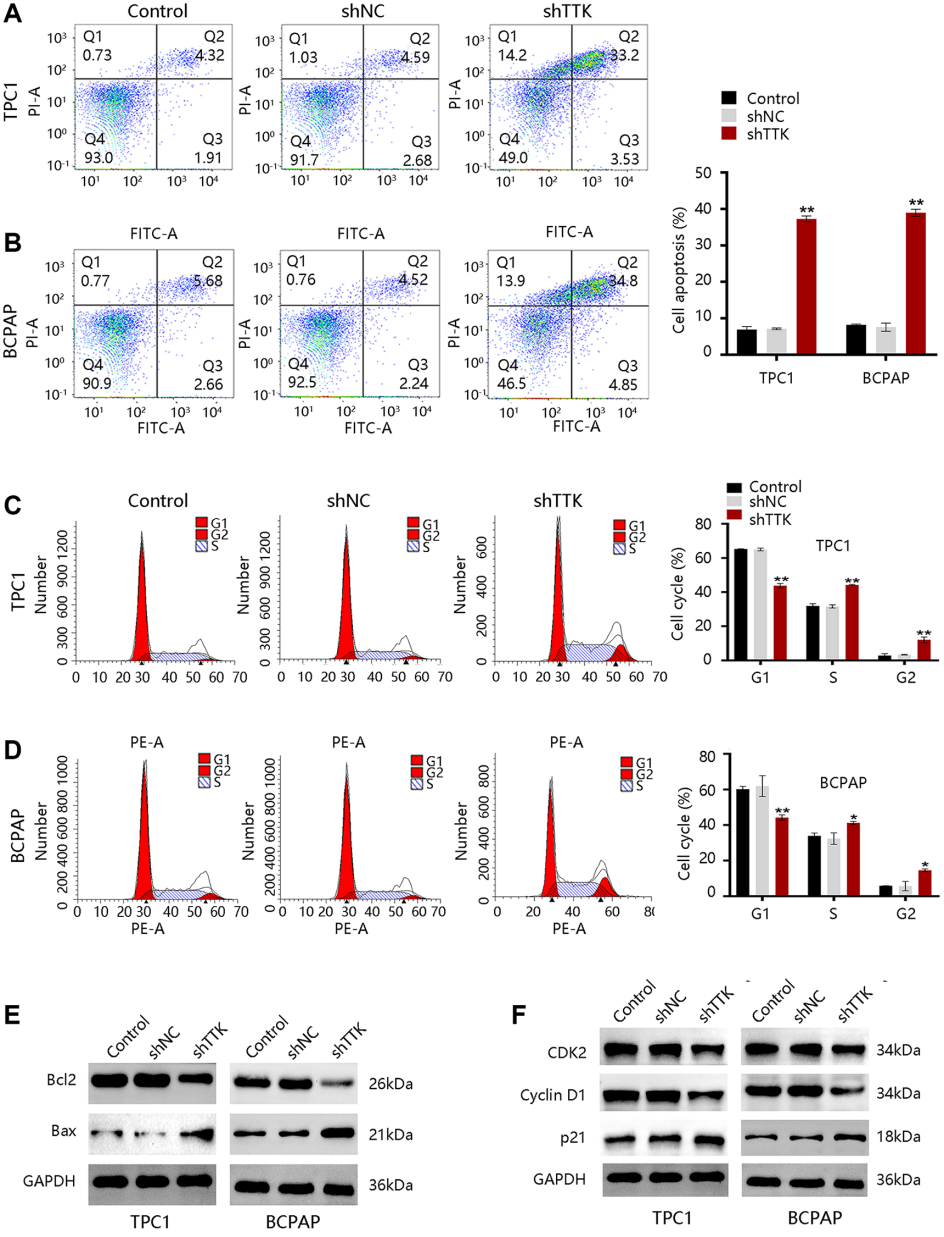
**TTK inhibits cell apoptosis and accelerates cell cycle progression in TPC1 and BCPAP cells.** (**A**, **B**) Results of cell apoptosis numbers (left) and cell apoptosis ratio (right). (**C**, **D**) Cell cycle results of peak diagrams (left) and cell distribution in different phases (right). Western blot for (**E**) apoptosis-related proteins and (**F**) cell cycle-related proteins in TTK-knockdown TPC1 and BCPAP cells. Compared with shNC, ^*^P < 0.05, ^**^P < 0.01.

To explore the mechanism of TTK regulating PTC cell growth, we performed analyses of cell apoptosis and cell cycle progression. TTK Knockdown increased TPC1 and BCPAP cell apoptosis (Figure 6A, 6B). Besides, the TTK-knockdown in two PTC cells significantly reduced G1-phase cells, while increasing S-phase and G2-phase cells compared to the control group (Figure 6C, 6D). Decreased Bcl2 protein expression but increased Bax protein expression further confirmed the apoptosis suppression by TTK (Figure 6E). Moreover, the expression of cell cycle-related proteins CDK2 and Cyclin D1 were downregulated and p21 was upregulated in TTK-knockdown TPC1 and BCPAP cells (Figure 6F). These results revealed that TTK might promote PTC cell growth by suppressing apoptosis and facilitating cell cycle progression.

### TTK contributes to cellular invasion and migration

**Figure 7:**
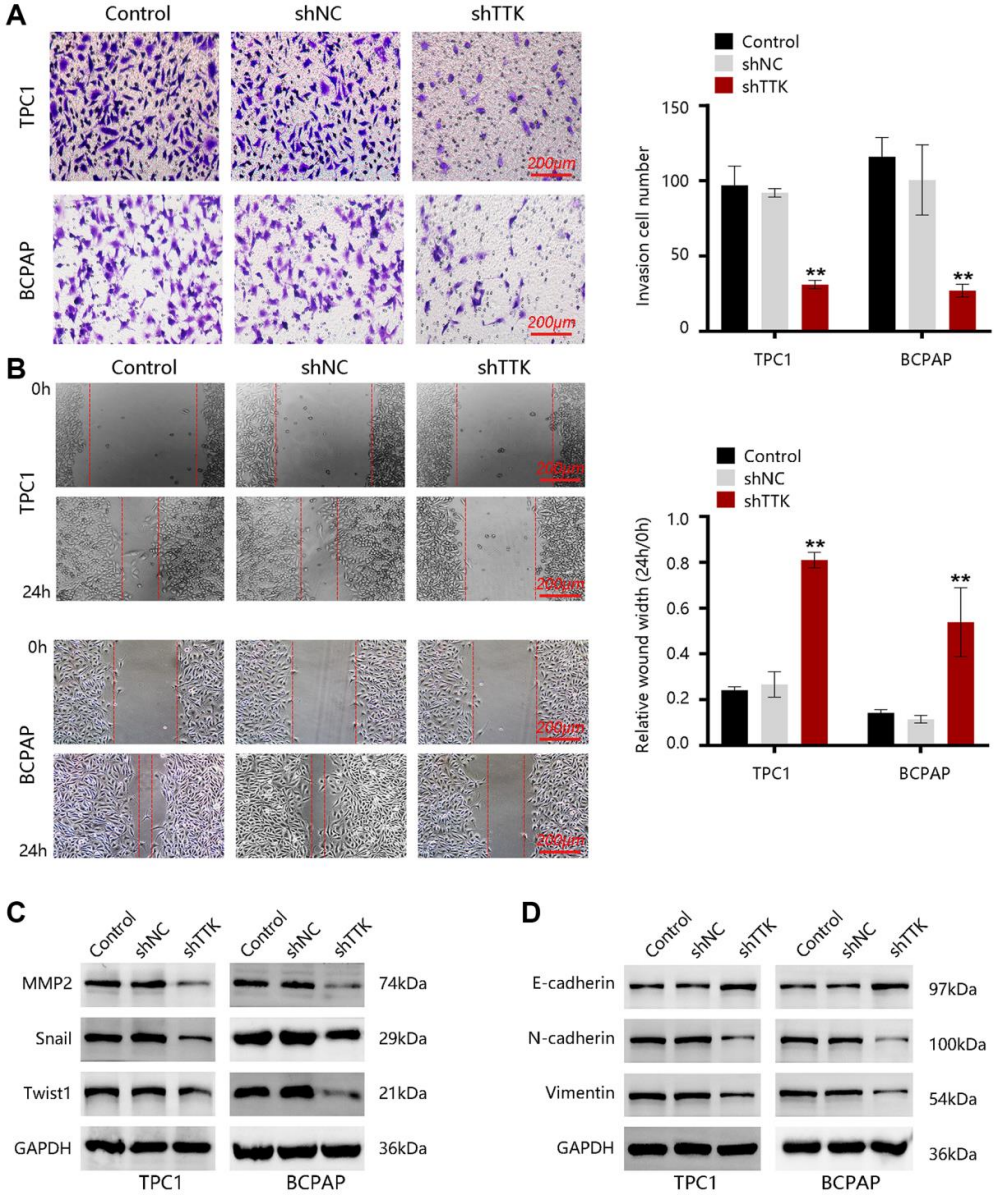
**TTK promotes PTC cell invasion and migration.** (**A**) The invasion of TPC1 and BCPAP cells transfected with shNC or shTTK and control for 24 h was measured by transwell invasion assay. (**B**) The migration of two PTC cells was assessed by wound-healing assay. Western blot for (**C**) metastasis-related proteins and (**D**) EMT maser genes in TTK-knockdown TPC1 and BCPAP cells. Compared with shNC, ^**^P < 0.01.

To investigate the effect of TTK in PTC metastasis, we carried out transwell and wound-healing assays. As shown in Figure 7A, the invasive capacity of PTC cells was significantly inhibited upon TTK knockdown. Besides, the migration of TPC1 and BCPAP cells was notably reduced once TTK was knocked down (Figure 7B). Then, the expressions of metastasis-related proteins MMP2, Snail, and Twist1 were examined. After TTK knockdown, the expressions of these proteins were downregulated (Figure 7C). These data indicated that TTK might be involved in PTC metastasis. Epithelial-mesenchymal transition (EMT) participates in cancer cell invasion and migration. To confirm whether TTK accelerates PTC cell invasion and migration via EMT, we evaluated the expression of “EMT master genes”. Upon TTK-knockdown, two PTC cells exhibited increased E-cadherin expression and decreased M-cadherin and Vimentin expressions (Figure 7D). Therefore, TTK might modulate cell invasion and migration by accelerating the EMT process and it might be involved in PTC metastasis.

### TTK activates the cell adhesion molecules (CAM) pathway

**Figure 8:**
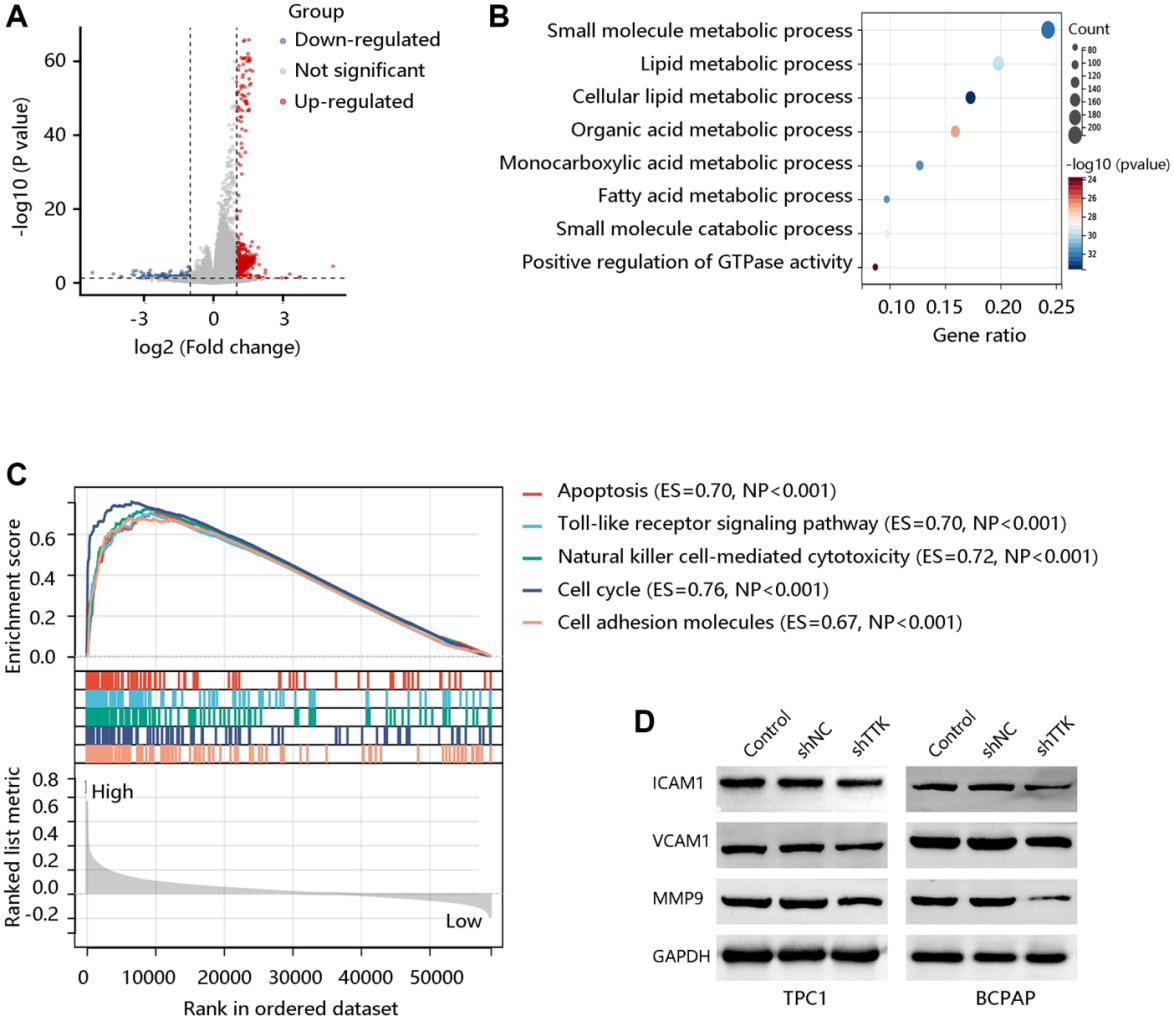
**TTK activates the cell adhesion molecules (CAM) pathway.** (**A**) The volcano plot shows differentially expressed genes (DEGs). (**B**) The biological processes of the significant DEGs. (**C**) Top five pathways enriched in high TTK expression group. (**D**) Western blot for the proteins in the CAM pathway.

To explore the potential signaling pathways regulated by TTK, we identified the DEGs between TTK-H and TTK-L (Figure 8A). We obtained 600 significant DEGs for analyzing the biological processes. As presented in Figure 8B, they mainly participated in the small molecule metabolic process, lipid metabolic process, and fatty acid metabolic process, which is essential in cancer cell migration and invasion. To meet the energy demands of cytoskeletal activity during migration, cells transport metabolic proteins and concentrate energy production to sites of high energy demand [27]. Subsequently, we performed GSEA and observed that the apoptosis, Toll-like receptor signaling pathway, natural killer cell-mediated cytotoxicity, cell cycle, and CAM were enriched in the TTK-H (Figure 8C).

Due to the potential value of TTK in metastasis, core proteins in the CAM signaling pathway were detected through western blotting. In the formation of metastasis, CAM plays a critical role in the adhesion of tumor cells to the endothelium. The protein expression of ICAM1, VCAM1, and MMP9 was decreased in TTK-downregulated TPC1 and BCPAP cells (Figure 8D). These findings suggested that TTK might promote PTC malignancy, especially its metastasis via activating the CAM signaling pathway.

### TTK was significantly associated with immune cell infiltration

**Figure 9:**
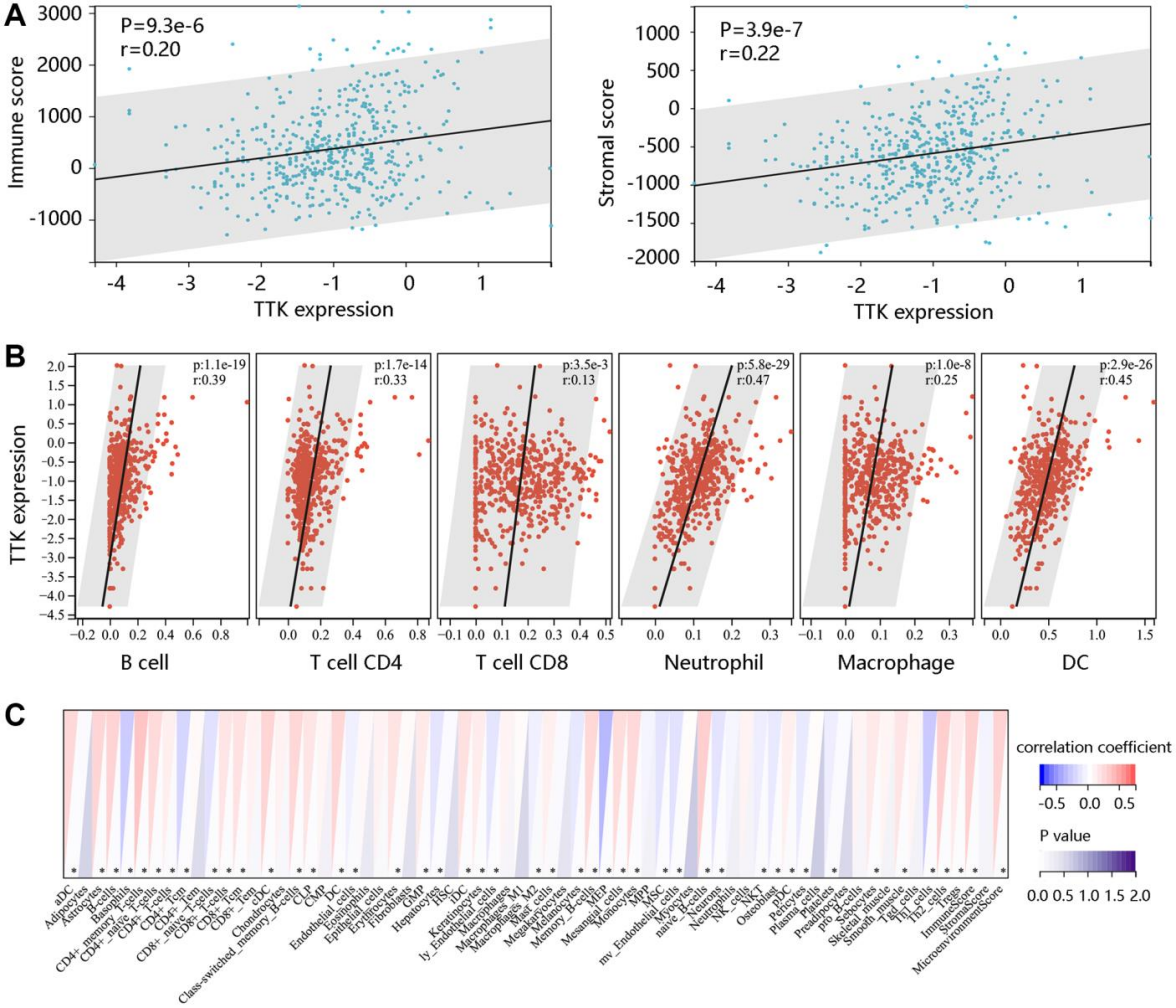
**The significant relationship between TTK and immune cell infiltration.** (**A**) TTK was positively related to immune and stromal scores using the ESTIMATE algorithm. The significant relation between TTK and several immune cells using the (**B**) TIMER and (**C**) xCELL algorithms.

To determine TTK involvement in the tumor immune microenvironment, we assessed the immune cell infiltrates using the ESTIMATE algorithm. A notable positive connection of TTK with immune- and stromal- scores was observed (*P* < 0.05) (Figure 9A). TIMER and xCELL algorithms confirmed that dendritic cells, T cell CD8, T cell CD4, and B cell were significantly linked to TTK (Figure 9B, 9C). The above findings suggested that TTK might influence immune cell infiltration levels in the PTC microenvironment.

### TTK is targeted by miR-582-5p

**Figure 10:**
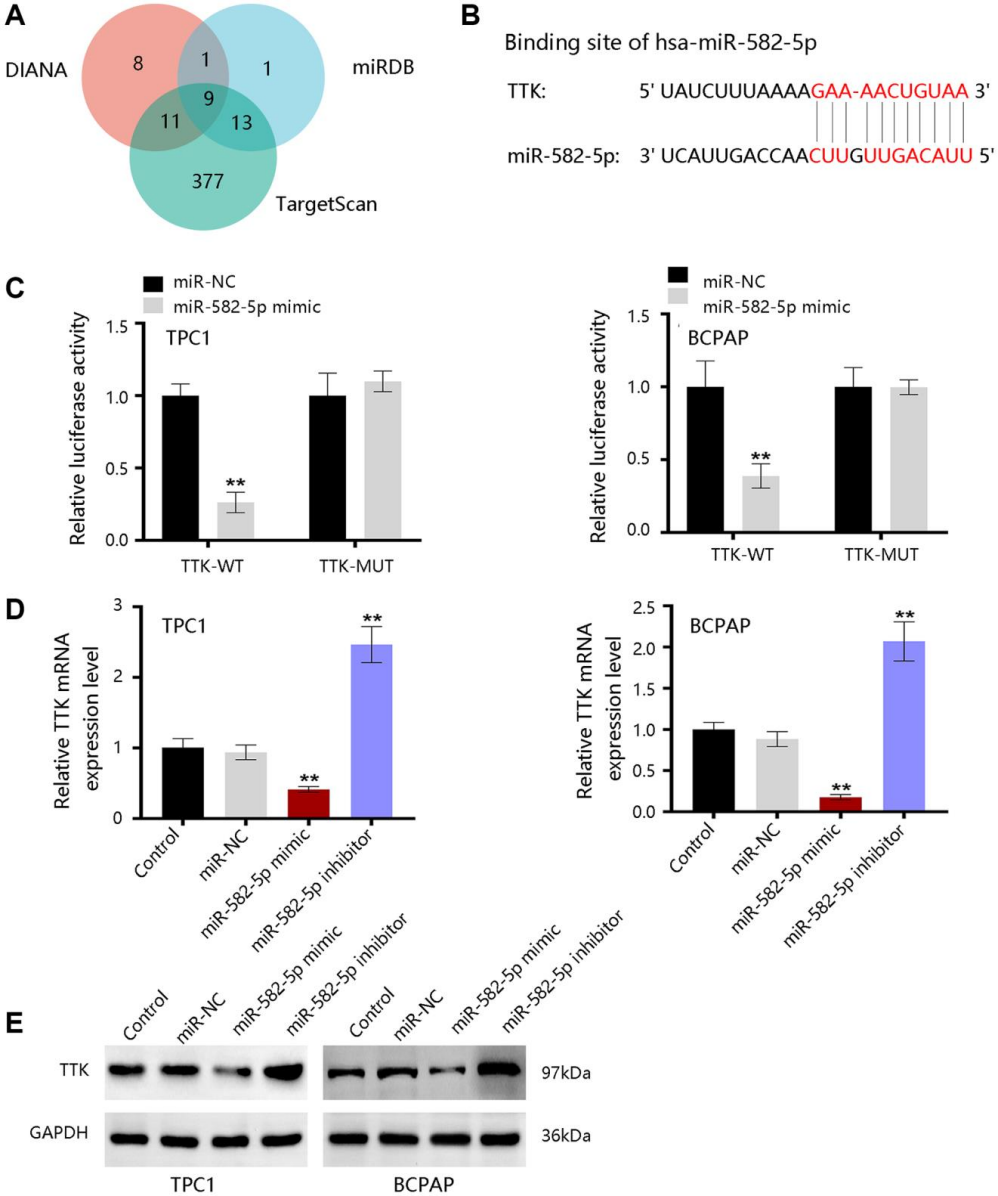
**The upstream miRNAs of TTK.** (**A**) Nine consistent miRNAs in three databases. (**B**) The binding site of miR-582-5p and TTK. (**C**) Validation of the binding site in TPC1 and BCPAP cells. (**D**) qRT-PCR and (**E**) western blot results for detecting expression change of TTK upon treatment of miR-582-5p mimics and inhibitors in PTC cells. Compared with miR-NC, ^**^P < 0.01.

To further reveal the underlying mechanism, bioinformatics tools were employed to predict the upstream miRNAs of TTK. There were 29, 24, and 410 miRNAs in DIANA, miRDB, and TargetScan databases, respectively (Figure 10A). Nine consistent miRNAs include miR-582-5p, miR-449b-3p, miR-376a-2-5p, miR-524-5p, miR-212-3p, miR-132-3p, miR-520d-5p, miR-4801, and miR-5697. We chose miR-582-5p as a potent regulator for further validation in PTC and the binding site was shown in Figure 10B. Luciferase activity was significantly inhibited by miR-582-5p in the wild-type reporter, while no change in the mutated reporter in two PTC cells (Figure 10C). Moreover, TTK expressions were downregulated upon miR-582-5p mimics transfection, while the expressions were upregulated upon miR-582-5p inhibitor transfection (Figure 10D, 10E). These results indicated that miR-582-5p negatively regulates the TTK expression.

## DISCUSSION

TTK is overexpressed in various cancers and serves as a target for the cancer treatment of colon, ovarian, breast, and glioblastoma. [28–30]. However, the role of TTK in PTC remains elucidated. Our study demonstrated high TTK expression in PTC and it had a certain value in predicting the DFI and PFI status in PTC. Besides, TTK overexpression independently predicted PTC prognosis and was notably related to PTC dedifferentiation. TTK participated in PTC cell proliferation, apoptosis, cell cycle, migration, and invasion. These results suggested the potential clinical value of TTK in PTC.

**Figure 11:**
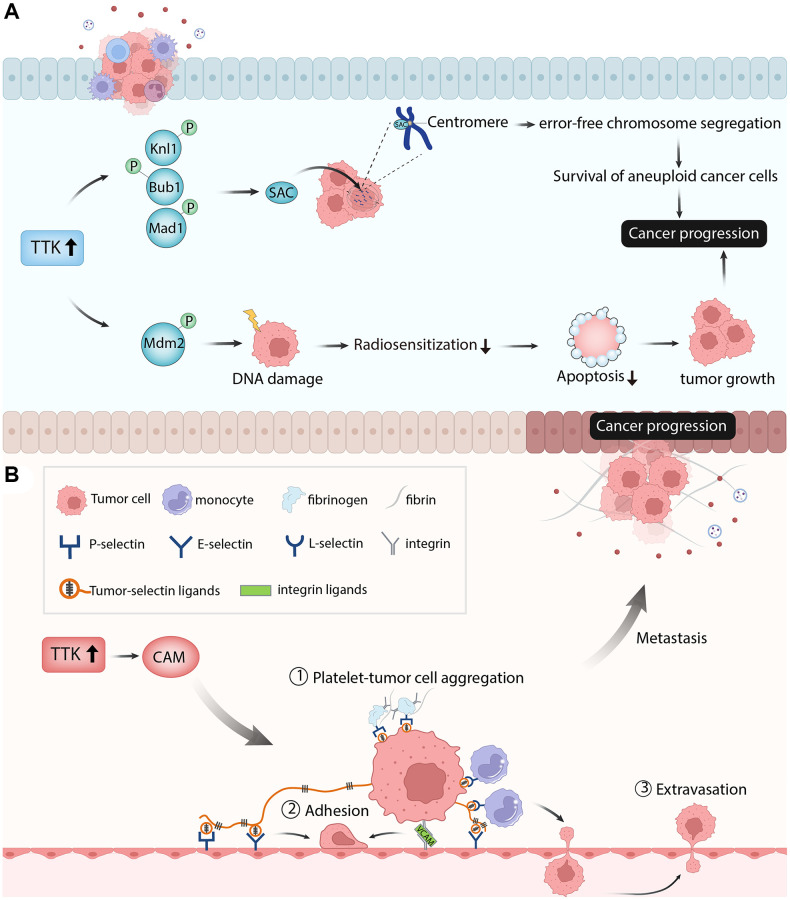
**The diagram showed the possible mechanism of TTK involved in papillary thyroid cancer.** (**A**) TTK was essential for tumor cell growth in the aspects of cell proliferation, cell cycle, and cell apoptosis. (**B**) TTK was related to cancer cell metastasis.

TTK plays a regulatory role in cancer cell proliferation, cell cycle, and apoptosis. Knockdown of TTK inhibited proliferation and increased apoptosis of gastric cancer cells through the Akt-mTOR pathway [31]. TTK depletion inhibited ovarian cancer cell proliferation by disturbing cell cycle progression [32]. In our study, PTC cell proliferation was suppressed, apoptosis was increased and the cell cycle was arrested after TTK was knockdown. The SAC is a conserved mitotic checkpoint that ensures accurate chromosome segregation during mitosis [33]. The inactivation of SAC in cancer leads to unequal segregation of chromosomes during mitosis, resulting in aberrant chromosomal numbers and cellular aneuploidy, hence enhancing chromosomal instability and the frequency of mitotic defects [34]. The protein kinase TTK is required for SAC activation [35]. Through Knl1, Bub1, and Mad1 phosphorylation sequentially, TTK can promote checkpoint activation and recruit checkpoint proteins to kinetochores in mitosis progress to ensure error-free chromosome segregation [36]. Elevated levels of TTK allow the survival of aneuploidy cancer cells and its overexpression has been observed in multiple cancers. TTK knockdown in glioblastoma and colorectal cancer cell lines reduced cell viability, led to abnormal cell progression and increased apoptosis [29, 37]. Similarly, the depletion of TTK decreased colonic survival and increased apoptosis in the pancreatic ductal adenocarcinoma cell line [14]. Therefore, the authors speculated that TTK overexpression might activate SAC through phosphorylating the substrates to promote cancer cell growth. In addition to the regulation of mitosis, TTK also facilitates DNA damage through phosphorylating Mdm2 [38]. DNA damage reduced radiosensitization and decreased cancer cell death, promoting cancer progression [39]. These findings revealed that TTK was essential for tumor cell growth in the aspects of proliferation, cycle, and apoptosis (Figure 11A).

Subsequently, we investigated the role of TTK in PTC cell migration and invasion using wound-healing and transwell assays. The migratory and invasive capacities of TPC1 and BCPAP were inhibited upon TTK knockdown. For validation, we detected the expression of metastasis-related proteins and “EMT master genes”, revealing that TTK might modulate cell migration and invasion through accelerating EMT in PTC cell lines and it might be related to PTC metastasis.

Next, we performed the enrichment analysis of the significant DEGs and found that they were mainly involved in metabolism-related processes, which is essential in cancer cell migration and invasion. The GSEA result showed that TTK mainly participated in apoptosis, Toll-like receptor signaling pathway, natural killer cell-mediated cytotoxicity, cell cycle, and cell adhesion molecules. Cell adhesion molecules include selections, cadherins, integrins, and others [40]. These molecules directly affect immune invasion and metastasis by mediating interactions with other cells and the extracellular matrix in the microenvironment [41]. Through fibrin and fibrinogen, P-selectin and integrins mediate the formation of Platelet-tumor cell aggregates. P-and E-selectins mediate the adhesion of tumor cells to the endothelium, while integrin and its interaction with VCAM-1 on tumor cells promote firm adhesion. Monocytes lead to tumor cell extravasation by facilitating tumor cell-endothelial interactions. This process depends on the conjugation of E-selectin and integrin [42]. Thus, the authors speculated that TTK might accelerate PTC cell metastasis by regulating the cell adhesion molecules pathway (Figure 11B). Due to the vital role of immune cells in tumor progression and metastasis, we performed an immune cell infiltration analysis and found that TTK was significantly correlated with several immune cells. The authors speculated that TTK might promote PTC development by influencing immune cell infiltration.

As short and highly conserved noncoding RNAs, miRNAs negatively regulate gene expression to modulate abundant biological processes. This study showed that miR-582-5p was an upstream regulator of TTK [43, 44]. Through its regulation of MTC proto-oncogene, aggressive phenotype would be suppressed in esophageal squamous cancer cells by miR-182 [45]. MiR-936 suppressed glioma progression by modulating ERBB4 [46]. Thus, the authors speculated that miR-582-5p might promote PTC progression by negatively regulating TTK expression.

For strengths, our study is novel in study design, which combines bioinformatics approaches with experiment verification. This is the first comprehensive research that systemically investigates the clinical significance of TTK in PTC and its overexpression promoting the PTC progression through accelerating cancer cell growth and metastasis. Moreover, this study reveals the possible mechanism of TTK involved in PTC. However, how the miR-582-5p regulates TTK expression to promote the PTC progression requires further investigation *in vitro*. The data from more independent cohorts will be applied for further analysis to validate the clinical value of TTK in PTC prognosis. The role of TTK in PTC dedifferentiation will be confirmed using *in vitro* experiments in the future.

In conclusion, TTK might be a potent regulator for tumor progression by the efficient bioinformatics approaches and validated by the *in vitro* experiments. Besides, TTK was significantly related to PTC dedifferentiation and immune cell infiltration. Finally, TTK expression can be modulated by miR-182-5p. These new findings indicated that TTK might be a potential biomarker and therapeutic target for PTC.
